# Retraction: Arthroscopic Meniscal Repair: University Hospital Southampton Experience

**DOI:** 10.7759/cureus.r135

**Published:** 2024-01-25

**Authors:** Nzubechukwu Ijezie, Hossam Fraig

**Affiliations:** 1 Surgery, Dorset County Hospital NHS Foundation Trust, Dorchester, GBR

This article has been retracted at the request of the authors because the article was submitted and published without undergoing formal review or receiving approval from the second author and others who were erroneously omitted from the author list. The authors greatly regret this error.

